# FSFI score and timing of tubal ligation in patients: preliminary results of an online survey

**DOI:** 10.1007/s00404-022-06547-8

**Published:** 2022-04-10

**Authors:** Steffen Walter, Mirjam Upadhjay, Jelena Beslic, Martin Pucher, Rebecca Herbel, Stavrou Stavroula, Davud Dayan, Wolfgang Janni, Florian Ebner

**Affiliations:** 1 University of Ulm, Ulm, Germany; 2 Frauenklinik Amperklinikum, Krankenhausstr. 15, 85221 Dachau, Germany; 3 IU Internationale Hochschule, Munich, Germany; 4 Gyn-Freising, Freising, Germany

**Keywords:** Tubal ligation, FSFI, Sterilisation, Regretting

## Abstract

**Introduction:**

Tubal ligation is the most common contraceptive method worldwide. Apart from a very low pearl index and anxiety relief, other benefits are not commonly recognised. In young patients, there is the post-operative risk of regretting the decision with the need for In-Vitro-Fertilisation or refertilising surgery. Positive side effects have not been widely published. In our study we investigated the change in the female sexual function index score after tubal ligation.

**Material and method:**

In this survey the FSFI score of participants around the time of the tubal ligation was compared with the FSFI score of intermediate and long-term time distance to the ligation.

**Results:**

The data indicate an increase in younger women seeking information on permanent contraception and whilst the FSFI score of the early group indicates a risk of female sexual dysfunction, the intermediate and long-term FSFI scores are comparable to published control groups.

**Discussion:**

Besides the obvious benefit of a low pearl index, tubal ligation may contribute to reduce the risk of female sexual dysfunction in the mid and long term. Informed consent is essential for the surgeon and patient to weigh up the risks and benefits individually including possible future perspectives on family planning.

## Introduction

Concerns about failure rates and side effects, as well as the male (un-)willingness to participate makes contraception a mainly female concern. The normal situation to request a TL is after family plans are completed [1]⁠. The definition of completed family plans varies by socioeconomic, cultural and political background. In recent years, younger females in the western world have increasingly expressed the wish to be childless by choice. This attitude has gained increasing attention in the popular press [2].⁠ The reasons for permanent contraception are various and it could be assumed that the resulting satisfaction with life has been reported to be positively correlated with a higher quality of life score and better perceived health [3]⁠.

Effective contraceptive methods cover a broad range and needs like barrier-, hormonal- and non-hormonal methods, permanent, short- or long-acting, oral, transdermal intake or implants or natural cycle monitoring. Each has its own benefits and side effects. As a permanent method with minimal or no hormonal side effects (e.g., early menopause), tubal ligation (TL) or vasectomy for the man is most commonly used. Despite the surgical risk of a TL being higher compared to a vasectomy due to the intraperitoneal or transuterine access way, still more women undergo the procedure than men [4–7]⁠. Tubal ligation can be achieved with various surgical techniques. Laparoscopic, open, and even vaginal approaches have been evaluated [6]⁠.

Besides the short-term surgical risks like infection, long-term follow-ups report of a “regretting” of the sterilisation. Regretting sterilisation has been reported in both women and men [8, 9]⁠. Patients under 30 years seem to be the major risk factor. For advice-seeking women, this results in the situation that they have the right for a TL by law, but it is nearly impossible to find a surgeon due to the possible long-term legal consequences [10, 11]⁠.

Further benefits like an improvement in quality of life, increased self-esteem or social life are not commonly known. The aim of this online survey was to compare the FSFI scores of patients at the time of TL (early ≙ ± 1 year of TL), 2–5 years after (intermediate) and 5–10 years (long-term) after TL.

## Material and method

To investigate the female sexuality, the FSFI is an evaluated tool in multiple languages and with a proven clinical relevance. The aim of this survey was to show an effect of a TL on the FSFI. Therefore, a three-armed study was created to compare the FSFI at the time of the TL (“early” group), sometime after (“intermediate” group) and after a long time (“long-term” group). Inclusion criterion for the “early” group were TL within ± 1 year. The “intermediate” group had a TL 2–3 years prior and the “long-term” group had a TL 4–10 years previously.

After ethics approval (University of Ulm #151/21) the German version of the FSFI and some baseline questions were published online and promoted via an association that supports a self-determined decision for tubal ligation webpage (www.selbstbestimmt-steril.de) and direct patient contact. While the association does not specifically target or cater to people who are and want to remain childfree-by-choice, it can be assumed that the majority of their followers would fall under this group, given that they have a large interest in preventing pregnancies with a secure and long-term method.

The survey was hosted according to data protection regulations on a survey webhosting service. The baseline questions are provided in the addendum in English.

Informed consent was obtained from the participants after logging into the website, with a short introduction where they were informed of the possibility to leave without answering any questions or at any stage during the 6-min survey. Participants chose a link depending on the time interval to the tubal ligation and proceeded with the survey. Variables were collected and stored at the webhost. The results were downloaded as excel files and further analysed.

Baseline variables are presented with descriptive statistics (median, standard deviation), and the FSFI scores were compared via Wilcoxon-test. A difference of 0.05 was selected.

The main FSFI questionnaire was identical between the subgroups and published literature. The baseline questions varied slightly depending on the subgroup to reflect the length of time since the TL. The questions are provided in the supplementary material. Collection of detailed socioeconomic information was not approved of by the ethics committee.

A recruitment number of *n* = 100 was aimed for each study arm.

## Results

The survey was accessible from 8th June, 2021 and results were retrieved on 6th November, 2021.

The total number of participants was 701. After excluding incomplete surveys, *n* = 441 remained for further analysis. These were distributed as follows: the “early” group had *n* = 249, the “intermediate” *n* = 177 and the “long-term” group *n* = 15.

The average age in the “early” and “long term” group were 28.6 years and 31.4 years, respectively. For the “intermediate” group, participants gave an age range. The predominant age group ‘aged’ according to the subgroup (i.e. “early” group 65% were under 26 years, “intermediate” group had 47% between 26 and 30 years and in the “long-term” group 50% were 31–35 years old).

The focus of the baseline questions in the early and long-term group were on the number of contraceptive methods, children, and well-being, whilst in the intermediate group the feelings after a TL were prioritised.

### Baseline “EARLY”

In the early group, 50% of participants had used 3 or more different contraceptive methods and 64% of the group agreed that the side effects contributed to their TL decision. The most common side effect was a reduced sexual desire (20%), followed by weight gain (8%) and irregular bleedings (7%) with 30% not answering the question and 33% not providing further details. The contraceptive method was found to be medium limiting by 40% and very limiting by 38%.

13% had “own” (biological) children and 85% were childless, with 82% never having had the need/feeling/urge for “own” children. 52% asked for the TL as family planning was completed.

72% were currently in a partnership, 10% were single, not looking for a partner and 15% were single, looking for a partner.

95% were not or only minorly influenced by the knowledge about the possibility of IVF after TL.

### Baseline “INTERMEDIATE”

72% of participants reported no or only minor physical limitation due to the TL. The stigma of a TL was found to be at least medium in 46% of participants. 82% felt emotionally relieved since the TL and 3 participants (1,4%) confirmed the wish for “own” children. 93% of the participants were “happy” in their current partnership status and 62% rated the quality of life with good or very good. The physical well-being had well or very well improved in 73%. Similarly, the psychological well-being had improved for 75% of participants by much or very much.

### Baseline “LONG-TERM”

In the long-term group, the time since the TL was given by 6 participants with up to 5 years, 5–10 years by 4 and by 5 participants it was longer than ten years ago. 8 participants had tried up to three contraceptive methods before TL and 6 gave the side effects as a contributing factor. The most important side effect was the reduced sexual desire (*n* = 3). The contraceptive method was found to be medium limiting by 8 and very limiting by 2 participants.

One participant had “own” (biological) children and the other 14 were childless, with 13 never having had the need/feeling/urge for “own” children. Seven asked for the TL after family planning was completed, 4 did not answer and another 4 answered “no”.

Thirteen were currently in a partnership and 2 were single, not looking for a partner.

The knowledge about the possibility of IVF after TL did not influence the decision in all participants.

### FSFI

The FSFI scores are provided for each subgroup in Table 1 including the control group of the primary FSFI evaluation by Rosen et al. [12]⁠. Due to the small numbers no statistical analysis was done with the “long-term” group. The overall score of the “early” group was significantly lower compared to the “intermediate” group (23,683 vs 29,433; *p* < 0.05). This difference was present over all questions and subdivisions of the questionnaire. The “intermediate” and “long-term” FSFI scores were similar to the control group (29.433 vs 29.257 vs 30.5).

**Table 1:** FSFI Scores per subgroup and external control group by Rosen et al. [13]; Baseline Survey questions

FSFI		Subgroup		Subgroup		Subgroup		Rosen et al.[13]
	*n* =	early	*n* =	intermediate	*n* =	long term	*n* =	Control
Desire		5.68		7.46		6.6	*131*	*6.9* ± *1.89*
Frequency	247	2.8 ± 0.28	176	3.68 ± 0.61	15	3.27 ± 0.5		*3.4* ± *1.04*
Level	248	2.88 ± 0.42	175	3.78 ± 0.67	15	3.33 ± 0.54		*3.5* ± *0.96*
Arousal		12.71		15.42		15.31	*130*	*16.8* ± *3.62*
Frequency	247	2.94 ± 0.8	176	3.49 ± 1.34	14	4 ± 0.29		*4.4* ± *1.06*
Level	240	3.3 ± 0.55	171	4.03 ± 0.82	14	3.64 ± 0.76		*4.0* ± *1.01*
Confidence	246	3.02 ± 0.58	172	3.54 ± 1.12	14	3.29 ± 0.86		*4.1* ± *1.06*
Satisfaction	233	3.45 ± 0.53	171	4.36 ± 1.12	13	4.38 ± 0.57		*4.4* ± *1.01*
Lubrication		15.18		18.05		17.76	*130*	*18.6* ± *3.17*
Frequency	234	3.94 ± 0.95	169	4.59 ± 1.48	13	4.54 ± 1.43		*4.6* ± *0.91*
Difficulty	246	3.86 ± 0.96	173	4.53 ± 1.47	14	4.5 ± 1.47		*4.7* ± *0.79*
Frequency of obtaining	243	3.62 ± 0.9	173	4.41 ± 1.49	14	4.29 ± 1.32		*4.6* ± *0.92*
Difficulty in	242	3.76 ± 0.93	172	4.52 ± 1.5	14	4.43 ± 1.33		*4.7* ± *0.79*
Orgasm		9.46		12.06		12.42	*129*	*12.7* ± *3.16*
Frequency	246	3.19 ± 0.56	174	3.98 ± 0.88	14	4.07 ± 0.93		*4.1* ± *1.21*
Difficulty	245	3.16 ± 0.54	173	4.12 ± 0.95	14	4.14 ± 0.98		*4.3* ± *1.11*
Satisfaction	244	3.11 ± 0.51	170	3.96 ± 0.97	14	4.21 ± 1.19		*4.4* ± *1.11*
Satisfaction		9.84		12.45		13.5	*130*	*12.8* ± *3.03*
With amount of closeness with partner	236	3.33 ± 0.69	173	4.08 ± 1.22	14	4.36 ± 1.47		*4.3* ± *1.12*
With a sexual relationship	218	3.39 ± 0.44	165	4.15 ± 1.05	14	4.71 ± 1.57		*4.2* ± *1.06*
With overall sex life	236	3.12 ± 0.37	169	4.22 ± 1.08	14	4.43 ± 1.25		*4.2* ± *1.11*
Pain		10.47		12.78		12.52	*130*	*13.9* ± *2.79*
Frequency during vaginal penetration	240	3.52 ± 0.9	172	4.27 ± 1.38	14	4.36 ± 1.47		*4.5* ± *1.09*
Frequency following vaginal penetration	239	3.58 ± 0.95	173	4.36 ± 1.43	13	4.08 ± 1.19		*4.7* ± *0.98*
Level during or following vaginal Penetration	224	3.37 ± 0.64	166	4.15 ± 1.13	13	4.08 ± 1.19		*4.7* ± *0.91*
Full scale		23.683		29.433		29.257	*129*	*30.5* ± *5.29*

## Discussion

[13, 14]⁠ These are mainly cross-sectional studies or case–control studies. These results identified several risk and contributing factors for sexual dysfunction. Several risk factors that can be influenced like cultural and socio-economic background [15, 16]⁠ seem to influence the FSFI. Other risk factors like age or chronic diseases are harder to overcome [17]⁠.

In our study the subgroup of females seeking permanent contraception was investigated in a cross sectional questionnaire. The average age and participant number of our study is within the age (23.5y–39.7y) and number (*n* = 51–2612) range of several FSFI studies [14, 18–22]⁠ investigating other subgroups.

Younger women seeking a tubal ligation face various hesitations from surgeons especially if childless. Goldhammer et al. [23]⁠ noted that women expected more information on contraception options or experienced discrimination based on their young age. The group of “childfree-by-choice” has been steadily increasing especially in females in their late twenties and gained popularity in the last decade [24]⁠. The age distribution of the participants in our survey supports this shift towards a younger age group interested in the topic of a TL. According to Goldhammer this age gap results in difficulties speaking about contraception or personal “discomfort”. These patients also noted a preconception by the doctor or had the feeling of prejudgment. This and other authors like Moore [25]⁠ confirm [10, 26]⁠ these perceptions from a clinical side. Unfortunately, these professional perceptions seem to be based on the association of young age as a risk factor for regretting the decision for TL [6, 27]⁠.

The regretting rate has been published with up to nearly 25% [27–31]⁠. Our results however indicate a much lower rate more in line with [32, 33]⁠ as only a minority of participants felt the need/urge for “own” children in the “intermediate” or “long-term” group. Low rates have also been reported in the subgroup of women without previous deliveries by Hillis et al. [30]⁠. This nullipara-subgroup made up the majority of our participants. Also, the knowledge of reversal possibilities had no or only little impact on the decision for a TL in our subgroup.

Our results confirm however the stigmatisation of advice seeking young women. Our participants indirectly support Sadatmahalleh et al. [21]⁠. Here stigmatisation can lead to a lower self-esteem. But the social and political situation in Iran and Germany are very different and the results should not be transferred without reflection. But in the western culture, previous studies with US college students also support these finding as realistic. College students rated childfree women as less warm than mothers on a five-point scale or expressed more moral concerns towards the childfree women or rated them lower on stereotypically positive traits for women [2]⁠. Publications regarding contraception counselling [23]⁠ or ethical issues [10, 26]⁠ of TL in young women reach similar conclusions. This indicates a stigmatisation which may lead to physical and psychological imbalance ultimately possibly resulting in sexual dysfunction. The interaction between physical and psychological well-being is well known for infertile couples seeking IVF and for TL patients [34, 35]⁠. The unanswered question is if this could be avoided by conserving or going through with a TL. For our study population, our data indicates an improvement with the TL in the short term. This needs further investigation to confirm the current literature [2, 25]⁠ and to confirm this in the long term group with a sufficient number of participants.

The main result of our study is the significant difference of the FSFI score in the ‘intermediate’ group compared to the ‘early’ group. The difference in those two groups is the time to TL and the FSFI score was nearly at a normal level in the early years after TL. In line with our survey, the baseline FSFI score has been repeatedly reported lower in patients with a TL compared to other subgroups ([21]⁠ TL-group 23.37 ± 4.99; [22]⁠ TL-group 2.43 ± 5.30, [36]⁠ TL group–19.63 ± 6.22 [14]⁠ intra gravidarum–17.5–23.2 depending on trimenon). But to the best of our knowledge no study group has correlated these scores over time to the TL surgery. Our results are in line with these findings regarding the level of the FSFI score and add a timeline to this defined subgroup. Based on these results the participants are at risk of developing female sexual dysfunction (FSD). FSD is estimated to be affecting up to 40% of women in the US [37]⁠ and treatment options are limited. Interestingly the results of our “intermediate” group show a significant increase in the FSFI score. This group reports a score that is in range of other “normal” subgroups ([21]⁠ non-TL group 26.07 ± 4.34; [22]⁠ condom group 28.03 ± 3.29; [36]⁠ control group 26.39 ± 7.39; [18]⁠ control group also Table 1). Though numbers are very low and, therefore, cannot be interpreted, the “long-term” group also reported an FSFI in this range.

The underpowered “long-term” group and a participation bias are the two most important weaknesses of our study and need to be addressed. Of course, participants recruited via a webpage that is assumed to mainly attract people who are childfree-by-choice are less likely to have regrets regarding the TL. The alternative to recruit from an outpatient clinic also bears a potential bias. Young patients requesting information on TL are more likely to fill out an anonymous online survey compared to a survey handed out at an appointment with a health professional who might not approve to the surgery. Whilst advice-seeking women can be found on webpages, providing further information the recruitment of long-term participants is more difficult. Accessing participants 5–10 years after the TL was difficult. This group may have a completely different social surrounding having accepted childlessness or a fulfilled family plan. These differente circumstances may shift the attention of the TL and the overcome problems. Currently the ‘mentoring’ for the next generation of women seeking advice is just evolving, with the intermediate group offering advice via web pages or social media. Women with a long-time TL may have lost touch with the “over-next” generation of women seeking advice on TL in younger years. A solution could be a multi-centre study and ideally this study would record the FSFI prospectively and other life circumstances over several years. But again, this network would have to offer an unbiased access and consulting to recruit unbiased. Which in return would mean that the topic would not lead to stigmatisation and would not influence the well-being of women. Further in our survey data on the education and social status of the participants are missing. These factors also influence the type of contraceptive methods and rate of regret [38–40]⁠ and should also be recorded in future surveys regarding this question.

## Conclusion

Women requesting a TL have a low FSFI score indicating a risk for the diagnosis of FSD. In our survey, the FSFI score was significantly higher after the TL. The underpowered “long-term” subgroup also showed improvements. The FSFI score of these subgroups were in a normal range. Our results may indicate to further–thus far unknown–future benefits of a TL and increase the complexity of counselling. A very well-informed consent with patient and care provider is essential for this procedure.
